# Testis‐specific hnRNP is expressed in colorectal cancer cells and accelerates cell growth mediating ZDHHC11 mRNA stabilization

**DOI:** 10.1002/cam4.4738

**Published:** 2022-04-05

**Authors:** Yuki Murakami, Hiroaki Konishi, Mikihiro Fujiya, Keitaro Takahashi, Katsuyoshi Ando, Nobuhiro Ueno, Shin Kashima, Kentaro Moriichi, Hiroki Tanabe, Toshikatsu Okumura

**Affiliations:** ^1^ Division of Metabolism and Biosystemic Science, Gastroenterology and Hematology/Oncology, Department of Medicine Asahikawa Medical University Asahikawa Japan; ^2^ Department of Gastroenterology and Advanced Medical Sciences Asahikawa Medical University Asahikawa Japan

**Keywords:** ATM, ATR, colorectal cancer, hnRNP G‐T, ZDHHC11

## Abstract

Various heterogeneous nuclear ribonucleoproteins (hnRNPs) have been reported to be associated with cancer cell growth. However, it remains unclear whether hnRNP G‐T, which is specifically expressed in the testis, is expressed in tumor cells, and whether hnRNP G‐T expressed in colorectal cancer (CRC) cells is associated with tumor progression. We herein report that hnRNP G‐T promoted cancer cell growth and stabilized mRNA of *ZDHHC11* in CRC. The cell growth was inhibited by transfection of siRNA of *hnRNP G‐T* in cancer cells, but not in non‐cancerous epithelial cells. The tumor promotive effect of hnRNP G‐T was confirmed in an HCT116 transplanted mouse model. RT‐PCR and western blotting indicated the augmentation of hnRNP G‐T in CRC in comparison to non‐cancerous cells. The downregulation of hnRNP G‐T inhibited cancer cell growth and promoted apoptosis in CRC. A transcriptome analysis combined with immunoprecipitation revealed that hnRNP G‐T stabilized 174 mRNAs, including *ZDHHC11* mRNA. The cell growth was also suppressed by the transfection of siRNA of *ZDHHC11* and the mRNA and the protein expression were decreased by the transfection of siRNA of *hnRNP G‐T*. These results suggested that hnRNP G‐T promotes the cell growth of CRC by regulating the mRNA of *ZDHHC11*. Therefore, hnRNP G‐T will be highlighted as an effective therapeutic target with less adverse effects in CRC therapy.

## Graphical abstract

hnRNP G‐T promoted cancer cell growth and mediated the stabilization of *ZDHHC11* mRNA in colorectal cancer cells. A growth inhibition effect induced by the reduction of hnRNP G‐T was detected in cancer cells, but not non‐cancerous cells. Thus, hnRNP G‐T is an attractive therapeutic target for colorectal cancer.

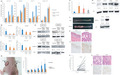

## 1 INTRODUCTION

Colorectal cancer (CRC) is a common cause of death worldwide.^1^ The prognosis of CRC has now been progressing by the development of molecular target therapy and the development of chemotherapy regimens. However, progressive CRC is still associated with a high rate of mortality; thus, a novel therapeutic target for CRC is desired.^2^

Posttranscriptional events, such as splicing, stabilization, and degradation of RNA, are an essential step for the development and differentiation for normal tissue. RNA binding proteins, such as heterogeneous nuclear ribonucleoproteins (hnRNPs), have essential roles in RNA regulation in normal cells and CRC cells.^3^, ^4^ For example, previous reports revealed that hnRNPA1 is augmented in CRC cells^5^ and that it stabilized oncogenic mRNAs, such as cyclin D1 and c‐Myc.^6^ We subsequently showed that hnRNP A1 was degraded when binding with microRNA‐18a, suppressing tumor progression through apoptosis in CRC cells.^7^ hnRNP K, the expression of which is correlated with the prognosis of CRC, stabilizes the mRNAs associated with cancer progression, including gastrin.^8^, ^9^ Likewise, we constructed siRNAs targeting 20 representative hnRNPs and discovered that all of these hnRNPs supported the progression of CRC.^10^ These investigations suggest that hnRNPs may be attractive therapeutic targets for CRC.

Heterogeneous ribonucleoprotein G, which is specifically expressed in the testis (*hnRNP G‐T: Gene symbol: RBMXL2*), is a member of the hnRNPs that plays essential role in spermiogenesis.^11^, ^12^ Ehrmann et al. showed that hnRNP G‐T prevents the mis‐splicing of mRNAs, is which associated with meiosis through the inhibition of splicing factors, such as Tra 2β, and binding with splice site sequences, thereby promotes the maturation of sperm.^13^ However, whether hnRNP G‐T is associated with disorders, including inflammatory and neoplastic diseases, has not been clarified. We previously demonstrated that hnRNP family molecules, such as hnRNP A0 and hnRNP H1, have pivotal functions by performing siRNA screening against 20 hnRNPs in colorectal cancer cells.^10^, ^14^ We also found—by siRNA screening—that hnRNP G‐T has tumor supportive functions. However, no reports have investigated the roles and mechanisms of hnRNP G‐T in the progression of CRC.

In the present study, we confirmed the tumor promotive effects of hnRNP G‐T, which was highly expressed in human CRC cell lines and tissues, but which had no effect in non‐tumorous cells. We also investigated the mechanism underlying the association between hnRNP G‐T and tumor progression in CRC cells.

## 2 MATERIALS AND METHODS

### 2.1 Cell culture

Human CRC cells (HCT116 and SW480) (American Type Culture Collection [ATCC]), human pancreatic cancer cells (SUIT‐2) (Health Science Research Resources Bank), and human colon epithelial cells (HCEC‐1CT) (Summit Pharmaceuticals International Corporation) were cultured in the appropriate medium^10^ in a humidified atmosphere with 5% CO_2_ at 37°C.

### 2.2 siRNA

siRNAs of *hnRNP G‐T* were obtained from Bioneer Inc. or Hokkaido System Science. The following siRNA sequences were generated to suppress the expression of *hnRNP G‐T*: sense siRNA of hnRNP G‐T #1, CAACAGUGGAAUACUAUAU (dTdT); anti‐sense, AUAUAGUAUUCCACUGUUG (dTdT); sense siRNA of #2, UUAGUUUCGUUGAAAAGGGAU (dTdT); anti‐sense, CCCUUUUCAACGAAACUAACA(dTdT).

### 2.3 Tissue samples of CRC

CRC specimens (*n* = 18) and adjacent normal tissues (*n* = 18) were obtained from 18 patients who received colonoscopy in our hospital. Total RNA was collected from the samples using an RNeasy mini kit (Qiagen) according to the manufacturer's instructions. This study was conducted with the understanding and written informed consent of all participants.

### 2.4 Western blotting

Cells were lysed in NP‐40 Cell Lysis buffer (ThermoFisher Scientific) containing cOmplete™ Protease Inhibitor Cocktail (Merck) and degenerated in Laemmli Sample Buffer containing 2‐mercaptoethanol at 95°C for 5 min. Equal amounts of protein were loaded onto SDS–PAGE (12.5%), transferred to a nitrocellulose membrane at 100 V for 60 min. Nonspecific binding of primary antibody was blocked using SuperBlock T‐20 (PBS; ThermoFisher Scientific) for 1 h. The following primary antibodies were diluted to 1:1000 in SuperBlock T‐20 (PBS) and incubated with membranes overnight at 4°C. The primary antibodies: hnRNPG‐T (Abcam; ab175228), ZDHHC11 (Abcam; ab69042), cleaved poly‐ADP‐ribose polymerase (PARP) (#5625, Cell Signaling Technology, Inc.), cleaved caspase‐3 (#96615625, Cell Signaling Technology), phosphor‐ATM (#5883, Cell Signaling Technology), ATM (#5883, Cell Signaling Technology), phosphor‐ATR (#30632, Cell Signaling Technology), ATR (#5883, Cell Signaling Technology), phosphor‐ERK (#4370, Cell Signaling Technologies), ERK (#5883, Cell Signaling Technology). Unbound primary antibody was removed in 0.05% Tween20‐PBS (T‐PBS), and the membranes were incubated with HRP‐conjugated secondary antibodies (R&D Systems, Inc.). The blots were visualized using a Super Signal West Pico enhanced chemiluminescence system (ThermoFisher Scientific) after washing with T‐PBS. The expression was Actin (612,656, BD Transduction Laboratories) was used for normalization of protein expression.

### 2.5 Real‐time PCR

Total RNA was isolated using a RNeasy mini kit (Qiagen) according to the manufacturer's instructions. cDNA was generated using a high‐capacity cDNA RT kit (ThermoFisher Scientific). The spectrum of the Ct value was monitored by an Applied Biosystems 7300 Real‐Time PCR system and using Taqman gene expression assays (HNRNPG‐T; Hs01059791, ZDHHC11; Hs00227137) in duplicate.

### 2.6 RNA‐immunoprecipitation

HCT116 cells were dissolved in NP‐40 cell lysis buffer (ThermoFisher Scientific) supplemented with a complete protease inhibitor cocktail (Merck) and RNasin (Promega Corporation). The cell lysates were clarified by centrifugation at 21,500× *g* for 10 min. The RNAs that form a complex with hnRNP G‐T were enriched using hnRNP G‐T antibody or isotype control with a Dynabeads immunoprecipitation kit (VERITAS Corporation). The RNAs were isolated from the precipitants with phenol–chloroform extraction and purified using a mirVana™ Isolation Kit (ThermoFisher Scientific). A whole transcriptome analysis using a Proton semiconductor sequencer was performed to exhaustively identify the mRNAs that bound with hnRNP G‐T. The binding of hnRNP G‐T and mRNAs was confirmed by RT‐qPCR.

### 2.7 Sulforhodamine B assay

The cells, which were treated with each siRNA by the reverse‐transfection method using RNAiMAX reagent, were plated in 96‐well microplates at 1.0 × 10^4^ cells/well. The Sulforhodamine B (SRB) assay was subsequentially performed according to the method reported by Vichai and Kirtikara.^15^

### 2.8 Xenografts

The posterior flank of 6–8‐week‐old male BALB/c nude mice was subcutaneously injected with HCT116 cells (2 × 10^6^ cells). *hnRNP G‐T* siRNA or scramble siRNA was administered daily with a GENOMONE‐Si transfection kit (Ishihara Sangyo, Co, Ltd.) into the transplanted tumor via local injection.

### 2.9 The mouse model of AOM–DSS carcinogenesis

BALB/c male mice (age: 6–8 weeks) were treated with azoxymethane (AOM; 10 mg/PBS/kg; Wako Pure Chemicals) via intraperitoneal injection, at 1 week after the injection of AOM, the mice were given 1% (wt/vol) dextran sulfate salt (DSS) (MP Biomedicals, LLC.) in drinking water for 1 week. Tumorous tissues were collected at day 63 and lysed in NP‐40 Cell Lysis Buffer.

### 2.10 Immunohistochemistry

After deparaffinization and rehydration, endogenous peroxidase activity was blocked with 0.6% H_2_O_2_ in ethanol for 25 min. The slides were then treated by the antigen‐retrieval technique with autoclaving in 10 mM citrate buffer (pH 9.0) for 20 min at 121°C. After blocking any nonspecific reaction with SuperBlock™ (PBS) Blocking Buffer (Thermo Fisher Scientific), the sections were incubated with anti‐hnRNP G‐T antibody (sc‐101,134; SantaCruz) at 4°C overnight. This step was followed by sequential incubation with the secondary antibody (ImmPRESS UNIVERSAL Reagent, Anti‐Mouse/Rabbit Ig) (VECTOR LABORATORIES, INC.) for 1 h at room temperature and stained using a DAB Peroxidase Substrate Kit, ImmPACT (VECTOR LABORATORIES). Nuclei were stained with hematoxylin.

### 2.11 Immunocytochemistry and TUNEL staining

HCT116 cells were plated on Lab‐Tek glass chamber slide (ThermoFisher Scientific) and were fixed with 4% paraformaldehyde overnight at 4°C. The cells were washed with PBS, permeabilized with 0.1% Triton X‐100, and blocked in 3% BSA in PBS. The slides were then sequentially incubated with primary antibodies (Ki‐67 [Novus, NB500‐170]) overnight at 4°C, washed with PBS, and then incubated with Alexa 488‐conjugated secondary antibodies (ThermoFisher Scientific) for 1 h at room temperature. The nuclei were counterstained with 4′,6‐Diamidine‐2′‐phenylindole dihydrochloride (Sigma Aldrich). TUNEL assays were performed with an In Situ Cell Death Detection Kit and TMR red (Roche Diagnostic) according to the manufacturer's instructions. The cells were mounted with an anti‐fade mounting medium, and the immunofluorescence was visualized using a fluorescence microscope (KEYENCE Corporation).

### 2.12 Transcriptome analyses using a Proton semiconductor sequencer

Transcriptome analyses using a Proton semiconductor sequencer were performed as previously described.^10^

### 2.13 Transcriptome analysis using a Novaseq 6000 sequencer

HCT116 cells were transfected with scramble or *ZDHHC11* siRNAs (*n* = 3). Agarose gel electrophoresis verified the RNA integrity and libraries were generated using a NEBNext Ultra II RNA Library Prep Kit (Illumina®). Sequencing was performed at Veritas Genetics using a Novaseq 6000 (Illumina®). An expression analysis of each sample was imported into the CLC Genomics Workbench software program (CLC bio). An unpaired *t*‐test determined the significance of the differences among the samples. The MetaCore software program performed the signal pathway and gene ontology (GO) analysis.

### 2.14 Actinomycin D chase experiment

HCT116 cells were transfected with scramble or *hnRNP G‐T* siRNAs (*n* = 3). After transfection for 24 h, the cells were treated with actinomycin D (2 μg/ml). Total RNA was extracted at the indicated time points (0, 2, and 6 h). *ZDHHC11* mRNA levels were analyzed by RT‐qPCR.

### 2.15 Plasmid DNA and transfection

HCT116 cells were transfected with control vector (pCMV6‐AC‐GFP vector), or *ZDHHC11* plasmid (ORIGENE, RG204383) and scramble or *hnRNP G‐T* siRNAs (*n* = 3) using Lipofectamine3000 reagent (ThermoFisher Scientific) according to the manufacturer's instructions.

### 2.16 Data assessment and presentation

The error bar and numbers show the SD. Significance was assessed by Student's *t*‐test and *p*‐values of <0.05 were considered to indicate statistical significance.

## 3 RESULTS

### 3.1 The knockdown of hnRNP G‐T exerted antitumor effects in CRC

**FIGURE 1:**
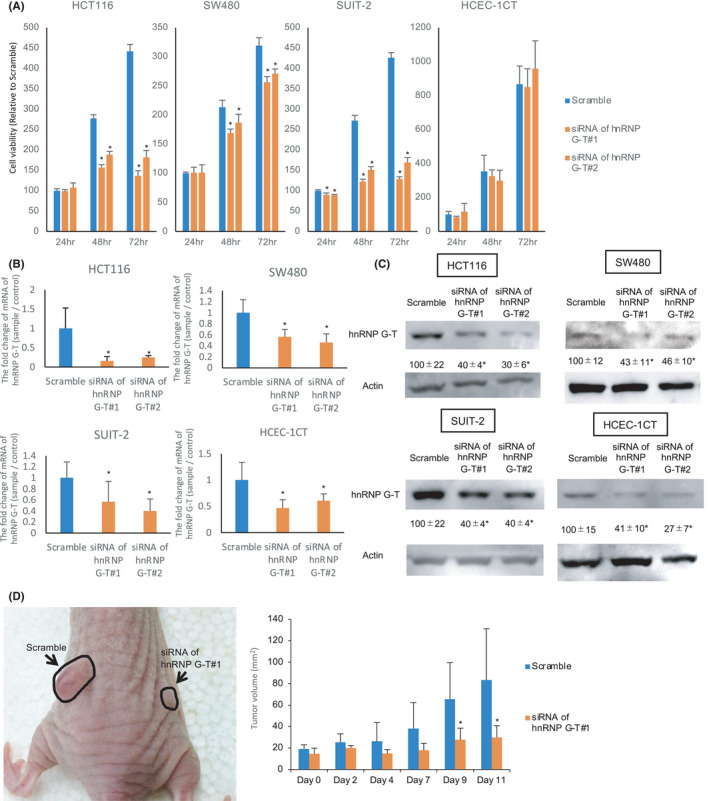
hnRNP G‐T promotes cell growth in cancer cells, but not non‐cancerous cells. (A) An SRB assay showed significant cell growth in cancer cells (HCT116, SW480, and SUIT‐2), but not non‐cancerous cells (HCEC‐1CT) (*n* = 5). (B, C) RT‐PCR (B) and western blotting (C) showed the knockdown efficacy of HCT116, SW480, Suit‐2, and HCEC‐1CT cells transfected with siRNA of *hnRNP G‐T* #1 and #2 at 48 h (*n* = 3). (D) The size of the HCT116 xenograft tumor was measured daily (n = 5)

First, to evaluate the tumor‐promoting effects of hnRNP G‐T, siRNA of *hnRNP G‐T #1* was transfected into non‐cancerous colorectal epithelial cells (HCEC‐1CT), CRC cells (HCT116 and SW480) and pancreatic cancer cells (SUIT‐2), and an SRB assay determined the cell growth (Figure 1A). A significant growth suppression effect was detected in cancer cells (HCT116, SUIT‐2, and SW480), but not in non‐cancerous cells (HCEC‐1CT), transfected with the siRNA of *hnRNP G‐T #1 and #2* in comparison to cells with scrambled RNA (Figure 1A). The cell growth was significantly reduced when two independent siRNAs of *hnRNP G‐T* were transfected into HCT116, SW480, and SUIT‐2 cells (the transfection efficacy is shown in Figure 1B, C), indicating that the growth changes were not caused by off‐target effects. siRNA of *hnRNP G‐T #1* was used for all subsequent experiments. To assess whether hnRNP G‐T has growth supportive functions in vivo, HCT116 cells were transplanted to the back of nude mice and siRNA of *hnRNP G‐T* was induced to the transplanted tumor. The tumor size of the *hnRNP G‐T* siRNA group was significantly smaller in comparison to the Scramble group (Figure 1D), suggesting that hnRNP G‐T has oncogenic properties in CRC cells in vivo.

### 3.2 hnRNP G‐T was augmented in CRC

**FIGURE 2:**
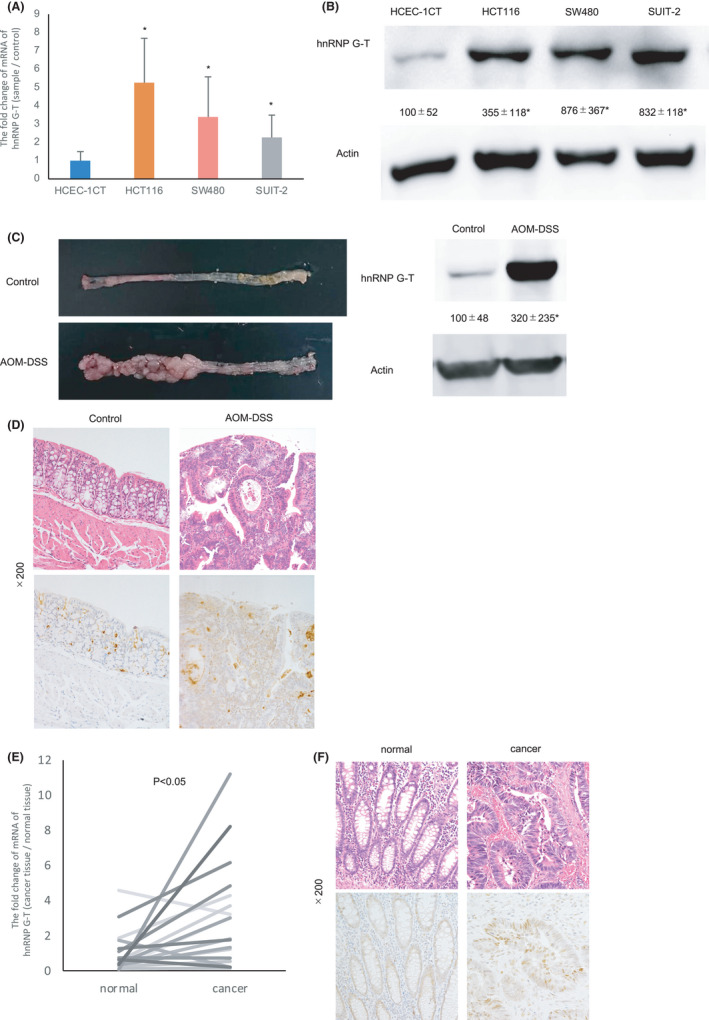
hnRNP G‐T is overexpressed in colorectal cancer (CRC). (A, B) RT‐PCR (A) and western blots (B) showed that hnRNP G‐T was augmented in HCT116, SW480, and SUIT‐2 cells in comparison to HCEC1CT cells (*n* = 3). (C) Western blotting showed that hnRNP G‐T was highly induced in the AOM‐DSS carcinogenesis model (*n* = 7–8). (D) Immunohistochemistry showed cancer cells positive for hnRNP G‐T in the AOM/DSS mouse model. (E) RT‐PCR showed the augmentation of *hnRNP G‐T* in CRC tissues in comparison to the normal epithelium (*n* = 18). (F) Immunohistochemistry showed cancer cells positive for hnRNP G‐T in human CRC tissue

To investigate whether hnRNP G‐T was highly induced in colorectal cells, the expression of hnRNP G‐T was compared between cancer and non‐cancerous cells. RT‐PCR and western blotting revealed the overexpression of *hnRNP G‐T* mRNA and protein in tumorous cells (HCT116, SW480, and SUIT‐2) in comparison to non‐tumorous cell (HCEC‐1CT) (Figure 2A,B). Western blotting showed that hnRNP G‐T is increased in the colorectal cancerous tissue of AOM/DSS carcinogenesis model in comparison to the normal colorectal epithelia of control mice (Figure 2C). Immunohistochemistry showed the augmentation of hnRNP G‐T in cancerous tissue of the AOM/DSS mouse model (Figure 2D). RT‐PCR using specimens obtained from 18 CRC tissues and 18 adjacent normal colorectal tissues showed that *hnRNP G‐T* mRNA was augmented in specimens of human cancerous lesions in comparison to non‐cancerous lesions (Figure 2E). Immunohistochemistry indicated that hnRNP G‐T is induced in human CRC tissue (Figure 2F). These suggested that hnRNP G‐T was aberrantly overexpressed in CRC.

### 3.3 hnRNP G‐T promoted cancer cell growth and inhibited apoptosis

**FIGURE 3:**
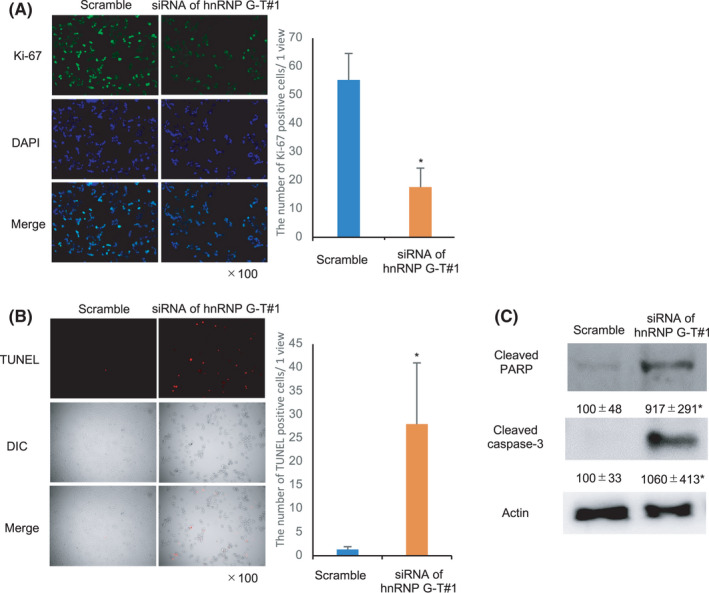
hnRNP G‐T promotes cell growth and inhibits apoptosis in colorectal cancer cells. (A) Immunocytochemistry showed that ki‐67 positive cells were reduced by the downregulation of hnRNP G‐T. (B) TUNEL staining showed the augmentation of TUNEL‐positive cells by the downregulation of hnRNP G‐T. (C) Western blots showed the induction of cleaved PARP and caspase‐3 in hnRNP G‐T‐knockdown cells

To investigate the influence of the downregulation of hnRNP G‐T on cell growth ability, immunocytochemistry of Ki‐67 and TUNEL staining were assessed. The Ki‐67‐positive cells was markedly decreased by *hnRNP G‐T* siRNA treatment (Figure 3A). The TUNEL‐positive cells in cells treated with siRNA of *hnRNP G‐T* were significantly higher in comparison to cells treated with scrambled RNA (Figure 3B). Likewise, western blotting of anti‐cleaved PARP and anti‐cleaved caspase‐3, which are parameters of apoptosis,^16^, ^17^, ^18^ showed that the expression of cleaved caspase‐3 and cleaved PARP were significantly augmented in *hnRNP G‐T*‐knockdown cells in comparison to control cells (Figure 3C). These data indicated that hnRNP G‐T promoted cell growth and inhibited apoptosis, and thereby exhibited oncogenic properties in CRC cells.

### 3.4 hnRNP G‐T upregulated *ZDHHC11* mRNA and promoted cell growth and *ZDHHC11* was highly expressed in CRC

**FIGURE 4:**
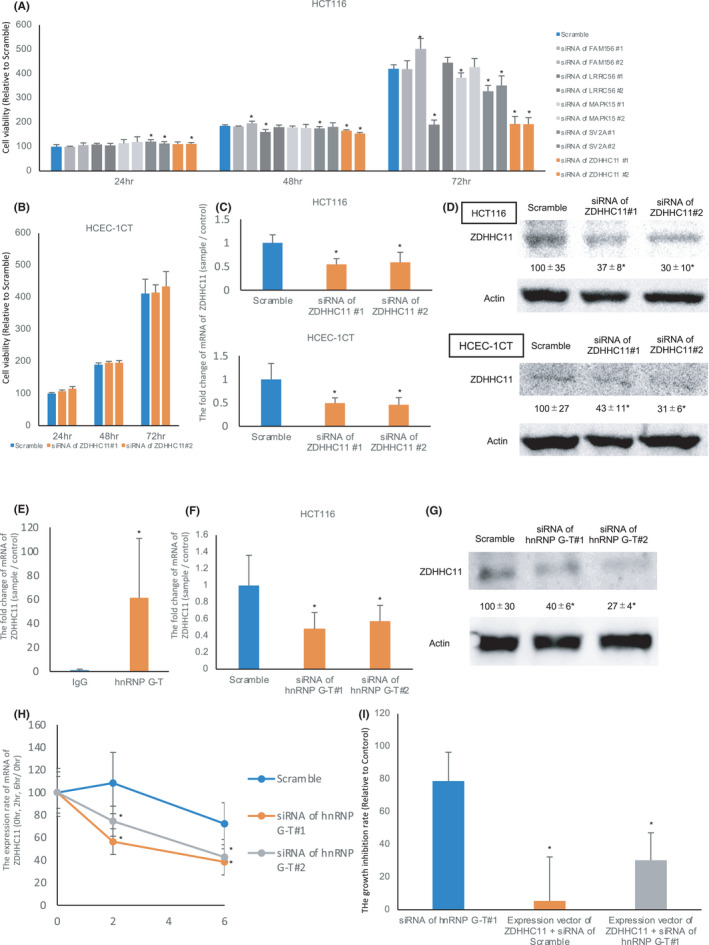
The expression of hnRNP G‐T is correlated with the expression of ZDHHC11 and ZDHHC11 was highly expressed in colorectal cancer. (A) An SRB assay showed the cell growth of HCT116 cells by the downregulation of the top five mRNAs. The downregulation of ZDHHC11 was associated with the strong inhibition of cell growth in HCT116 cells (*n* = 5). (B) The downregulation of ZDHHC11 showed no suppression of cell growth in HCEC‐1CT cells (*n* = 5). (C, D) RT‐PCR (C) and western blotting (D) showed the knockdown efficacy of HCT116 and HCEC‐1CT cells transfected with siRNA of *ZDHHC11* at 24 h (*n* = 3). (E) RNA‐immunoprecipitation combined with RT‐PCR confirmed the direct binding of hnRNP G‐T and *ZDHHC11* mRNA (*n* = 3) (C). F, G RT‐PCR (F) and western blots (G) showed that the *ZDHHC11* mRNA and protein expression were decreased in hnRNP G‐T‐downregulated HCT116 cells (n = 3). (H) The actinomycin D experiment showed that hnRNP G‐T stabilized the *ZDHHC11* mRNA in HCT116 cells (*n* = 3). (I) The growth inhibition rate calculated by the SRB assay showed that growth suppression induced by hnRNP G‐T downregulation recovered with the overexpression of ZDHHC11. The growth inhibition rate (%) = [1 − (OD510 nm of each of the samples on day 3––OD510 nm of each of the samples on day 1)/(OD510 nm of Control on day 3––OD510 nm of Control on day 1)] × 100

To reveal mRNAs bound with hnRNP G‐T in CRC cells, RNA‐immunoprecipitation using hnRNP G‐T antibody combined with a whole transcriptome analysis was performed in HCT116 cells. The transcriptome analysis with immunoprecipitated RNAs showed that 3212 mRNAs bound directly to hnRNP G‐T (value of fold change; > 4, *p* < 0.01) (Table S1). To identify mRNAs regulated by hnRNP G‐T, total RNA was collected from hnRNP G‐T knockdown cells, and then a transcriptome analysis was performed. This showed that 1941 mRNAs were significantly decreased in hnRNP G‐T‐downregulated cells (absolute value of fold change, >4, *p* < 0.01) (Table S2). RNA‐immunoprecipitation using hnRNP G‐T antibody combined with a transcriptome analysis and a transcriptome analysis using hnRNP G‐T knockdown cells suggested that the expression of 223 mRNAs was directly regulated by hnRNP G‐T. The molecular functions of these 223 mRNAs were then searched by a GO analysis. One hundred seventy‐four genes, the functions of which have already been reported, were selected for the following experiments (Table S3). In the 174 genes, the top five mRNAs of the IP‐transcriptome assay (*FAM156*, *LRRC56*, *MAPK15*, *SV2A*, and *ZDHHC11*) were subsequently evaluated. To evaluate whether hnRNP G‐T promoted tumor cell growth mediating the five mRNAs, these mRNAs were knocked down with the siRNA for each mRNA and cell growth was assessed. The SRB assay showed strong growth inhibition in HCT116 cells transfected with the siRNAs of both *ZDHHC11 (Gene symbol: ZDHHC11) #1 and #2* in comparison to cells with scrambled RNA (Figure 4A). In contrast, the downregulation of ZDHHC11 had no effect on the cell growth in non‐tumorous HCEC‐1CT cells (Figure 4B). The knockdown efficacy of siRNA of *ZDHHC11* of each cell is shown in Figure 4C,D. RNA‐immunoprecipitation combined with RT‐PCR confirmed the direct binding of hnRNP G‐T and *ZDHHC11* mRNA in HCT116 cells (Figure 4E). To reveal whether hnRNP G‐T stabilized *ZDHHC11* mRNA directly, the expression of ZDHHC11 was assessed in hnRNP G‐T‐downregulated cells. RT‐PCR and western blotting showed that the mRNA and protein expression of ZDHHC11 was decreased in HCT116 cells and HCEC‐1CT cells transfected with siRNA of *hnRNP G‐T* (Figure 4F,G, Figure S1A,B). To confirm the *ZDHHC11* mRNA stabilization by hnRNP G‐T, actinomycin D (which inhibits the generation of mRNA) was applied on hnRNP G‐T downregulated cells. RT‐PCR showed the downregulation of *ZDHHC11* in actinomycin D treated hnRNP G‐T downregulated cells (Figure 4H). To assess whether tumor cell growth was recovered by the overexpression of ZDHHC11 in hnRNP G‐T downregulated cells, the expression vector of *ZHDHHC11* and siRNA of *hnRNP G‐T* were co‐transfected to HCT116 cells. An SRB assay revealed that the growth suppression mediating hnRNP G‐T downregulation was almost recovered by the overexpression of ZDHHC11 (Figure 4I, the expressional analysis of ZDHHC11 is shown in Figure S2A). These data suggest that hnRNP G‐T stabilized the *ZDHHC11* mRNA, and thereby promoted the progression of CRC.

**FIGURE 5:**
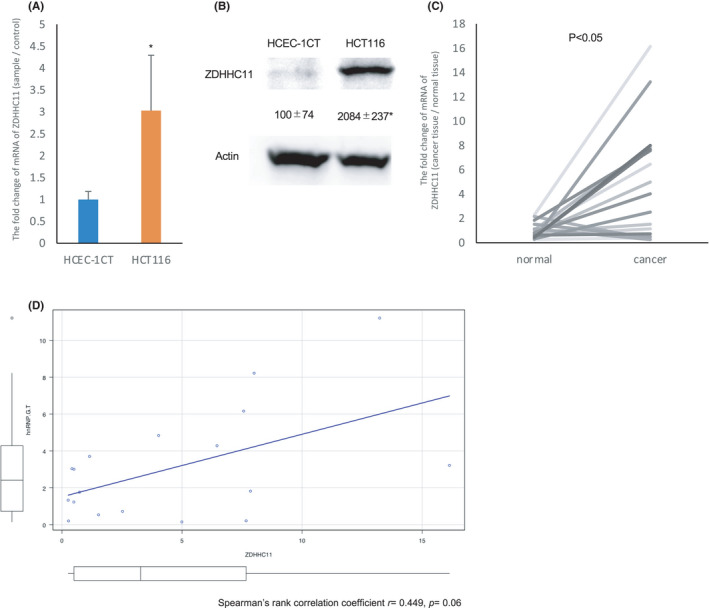
ZDHHC11 is highly induced in colorectal cancer (CRC). (A, B) RT‐PCR (A) and western blots (B) showed that the *ZDHHC11* mRNA and protein expression were decreased in hnRNP G‐T‐downregulated HCTEC‐1CT cells (*n* = 3). (C) RT‐PCR showed the augmentation of *ZDHHC11* in CRC tissues in comparison to the normal epithelium (*n* = 18). (D) The mRNA expression of *ZDHHC11* tends to correlate with the expression of *hnRNP G‐T* (*n* = 18)

To assess the abnormal expression of ZDHHC11 in colorectal cells, the expression of ZDHHC11 was compared between cancer and non‐cancerous cells. RT‐PCR and western blotting showed the overexpression of the mRNA and protein of ZDHHC11 in tumorous cells (HCT116) in comparison to non‐tumorous cell (HCEC‐1CT) (Figure 5A,B). Furthermore, the overexpression of *ZDHHC11* mRNA (in comparison to non‐cancerous lesions) was confirmed in specimens of human cancerous lesions (Figure 5C). To investigate the correlation of the *hnRNP G‐T* and *ZDHHC11* expression, we compared the expressional profiles of these two genes. The correlation coefficient was 0.449 (*p* = 0.06, *n* = 18) (Figure 5D), suggesting that the expression of *hnRNP G‐T* tends to be correlated with the expression of *ZDHHC11* mRNA in CRC.

### 3.5 ZDHHC11‐promoted cell growth and inhibited apoptosis in CRC

**FIGURE 6:**
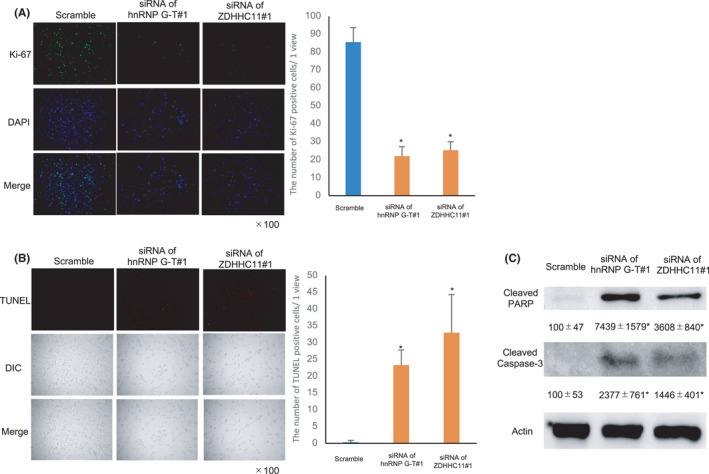
ZDHHC11‐promoted cell growth and inhibited apoptosis in colorectal cancer. (A) Immunocytochemistry showed that ki‐67‐positive cells were reduced by the downregulation of hnRNP G‐T or ZDHHC11. (B) TUNEL staining showed that TUNEL‐positive cells were augmented by the downregulation of hnRNP G‐T or ZDHHC11. (C) Western blots showed the induction of cleaved PARP and cleaved caspase‐3 in hnRNP G‐T or ZDHHC11‐knockdown cells (*n* = 3)

To confirm the cell proliferative and antiapoptotic functions of ZDHHC11, Ki‐67 staining, and TUNEL assay were performed. The number of Ki‐67‐positive cells were markedly decreased with *hnRNP G‐T* or *ZDHHC11* siRNA treatment (Figure 6A) and the number of TUNEL‐positive cells in cells treated with *hnRNP G‐T* or *ZDHHC11* siRNA were significantly higher in comparison to cells treated with scrambled RNA (Figure 6B). Western blotting showed that the expression of cleaved caspase‐3 and cleaved PARP was significantly augmented in hnRNP G‐T‐ or ZDHHC11‐downregulated cells (Figure 6C). These data indicate that ZDHHC11 promoted cell growth and inhibited apoptosis in CRC.

### 3.6 hnRNP G‐T‐ZDHHC11‐promoted cell growth by activating the ATM/ATR pathway and ERK1/2 in CRC

**FIGURE 7:**
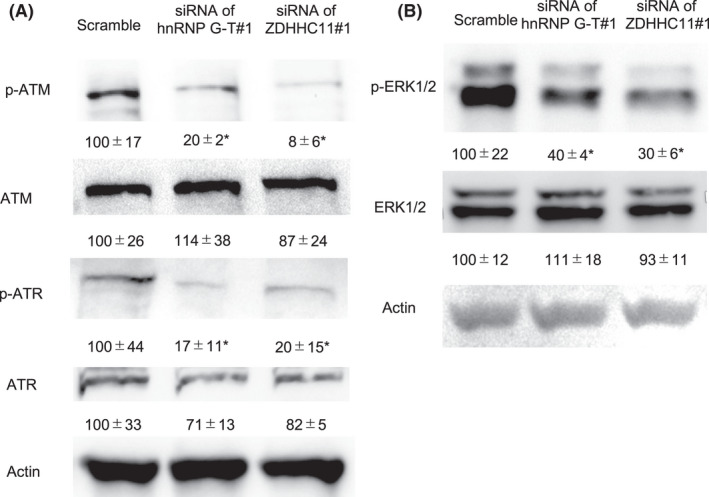
hnRNP G‐T‐*ZDHHC11*‐promoted cell growth by activating the ATM/ATR pathway and ERK1/2 in colorectal cancer. (A, B) Western blots showed that deactivation of phosphorylated ATM and ATR (A) in hnRNP G‐T or ZDHHC11‐knockdown cells (*n* = 3) and the deactivation of phosphorylated ERK (B) in hnRNP G‐T or ZDHHC11‐knockdown cells (*n* = 3)

To investigate the tumor‐promotive mechanism of ZDHHC11 in CRC cells, we performed a transcriptome analysis in ZDHHC11‐downregulated HCT116 cells. One thousand eighty‐seven mRNAs were aberrantly expressed in ZDHHC11‐downregulated cells (absolute value of fold change; >4, *p* < 0.05) (Table S4). A signal pathway analysis indicated that the downregulation of ZDHHC11 in CRC was closely associated with the ATM/ATR pathway (Tables S5 and S6). To confirm whether hnRNP G‐T regulates the ATM/ATR pathway through the stabilization of *ZDHHC11* mRNA, the phosphorylation of ATM and ATR was assessed in hnRNP G‐T‐ or ZDHHC11‐knockdown CRC cells. Western blotting showed that dephosphorylation of ATM and ATR, but not their whole expression, was induced by the knockdown of hnRNP G‐T or ZDHHC11 in HCT116 cells (Figure 7A). Previous investigations showed that the ATM/ATR pathway was positively regulated by ERK signal transduction.^19^, ^20^ Western blotting showed the downregulation of phosphorylated ERK1/2 by the knockdown of hnRNP G‐T or ZDHHC11 in HCT116 cells (Figure 7B). These findings suggest that hnRNP G‐T activates the ATM/ATR/ERK pathway through the stabilization of *ZDHHC11* mRNA, and thereby promotes the progression of CRC.

## 4 DISCUSSION

In this study, we demonstrated that hnRNP G‐T, which is specifically expressed in the testis, was induced in CRC cells and that the inhibition of hnRNP G‐T had a tumor suppressive effect. Likewise, hnRNP G‐T supported cell growth and inhibited apoptosis by the direct stabilization of the tumor promotive *ZDHHC11* mRNA, and thereby promoted the progression of CRC. Importantly, the expression of *hnRNP G‐T* mRNA and protein was correlated with the *ZDHHC11* mRNA and protein expression (Figure 4D,E), and the growth of non‐cancerous cells was not inhibited by the downregulation of hnRNP G‐T. Therefore, hnRNP G‐T may be an attractive therapeutic target for CRC.

Our SRB assay and xenograft study showed that the downregulation of hnRNP G‐T significantly decreased the proliferation of cancer cells, but not non‐cancerous cells. We demonstrated that hnRNP G‐T, which is normally specifically expressed in the testis, is augmented in cancer cells and works as an oncogene. To utilize hnRNP G‐T as a cancer treatment target, the toxicity of germ cells should be monitored because CRC develops in young as well as old population.

We demonstrated that hnRNP G‐T bound directly to and stabilized *ZDHHC11* mRNA, thereby promoted the CRC cell growth. ZDHHC11, a poorly characterized member of the palmitoyltransferase family [Human DHHC proteins: A spotlight on the hidden player of palmitoylation], is considered to be associate with the progression of several types of cancer, and the gain of the ZDHHC11 coding region (5p15.33) in bladder cancer and non‐small cell lung cancer, and *ZDHHC11* mRNA is targeted by the tumor suppressor microRNA, miR‐150, in Burkitt lymphoma.^21^, ^22^, ^23^ However, the association of ZDHHC11 with CRC and the mechanism underlying its tumor proliferative functions have not been reported. Our SRB assay confirmed the inhibition of cell growth in HCT116 cells, but not in HCEC‐1CT cells, by the knockdown of ZDHHC11. Interestingly, *ZDHHC11* mRNA expresses numerous cells, including gastrointestinal tract and testis whereas, the expression of *hnRNP G‐T* mRNA and protein was testis specific^24^ (The expression data downloaded from Human Protein Atlas [www.proteinatlas.org] was added to Figure S5 and S6). These data suggest that hnRNP G‐T‐*ZDHHC11* mRNA pathway works specifically in cancer cells and may be an attractive therapeutic target for CRC treatment.

It is well known that the ATM/ATR pathway is a key mediator of the DNA damage response (DDR), which regulates the cell functions, including the cell cycle and apoptosis,.^25^ Recently, ATM and ATR inhibitors are highlighted as antitumor agents because numerous cancers, including CRC, utilize the ATM/ATR‐associated DDR for survival and drug resistance.^26^, ^27^, ^28^, ^29^, ^30^ Interestingly, we showed that the downregulation of the hnRNP G‐T‐*ZDHHC11* mRNA pathway, which was highlighted as upstream signaling of the ATM/ATR pathway by our transcriptome analysis, inhibited the growth suppression effect in cancer cells, but not in non‐cancerous cells, suggesting that the antitumor effects of ATM/ATR signaling‐targeted therapy may be achieved by targeting hnRNP G‐T or ZDHHC11, with fewer adverse events.

The RAS–RAF–MEK–ERK signal pathway is regarded as an important therapeutic target in CRC and has been widely studied.^31^, ^32^ Previous reports showed that ERK signal transduction enhanced the ATM/ATR pathway, whereas ERK activation is also induced by DNA damage‐induced checkpoint activation, including the ATM/ATR pathway.^33^, ^34^, ^35^ Our western blotting experiment showed that ERK signaling as well as ATM/ATR signaling was attenuated by the downregulation of hnRNP G‐T and ZDHHC11, supporting that the hnRNP G‐T‐*ZDHHC11* pathway promoted tumor progression, which mediated the ATM/ATR signaling pathway. However, how ZDHHC11 attenuates ATM/ATR/ERK signaling remains to be elucidated, because ZDHHC11 is a member of the palmitoyltransferase family molecules which are not kinases. The molecular mechanism underlying the phosphorylation of ATM/ATR/ERK, which mediates ZDHHC11, should be investigated for the hnRNP G‐T‐*ZDHHC11* pathway to be used as a therapeutic target in CRC.

The cleavage of PARP and caspase‐3 was strongly induced by the downregulation of hnRNP G‐T in comparison to the downregulation of ZDHHC11. An IP‐transcriptome analysis revealed that hnRNP G‐T bound with and regulated numerous mRNAs, including apoptosis‐related mRNAs, as well as *ZDHHC11* mRNA, suggesting that the cancer progression pathway, which mainly regulates apoptosis, is regulated by hnRNP G‐T in CRC cells. We also demonstrated that 223 mRNAs directly bound to hnRNP G‐T. In the present study, we assessed the tumor promotive function of the top five mRNAs identified by RNA‐immunoprecipitation and a whole transcriptome analysis. However, tumor promotive mRNAs, such as cyclin‐dependent kinase, were also stabilized by hnRNP G‐T and can be associated with tumor cell growth. In fact, in HCT116 cells, the cleavage of caspase 3 and PARP was strongly induced by hnRNP G‐T downregulation in comparison to ZDHHC11 downregulation, suggesting that hnRNP G‐T promotes the progression of CRC through the stabilization of several oncogenic mRNAs. Notably, we demonstrated that cell growth suppression of CRC mediating the downregulation of hnRNP G‐T almost recovered with the overexpression of ZDHHC11, suggesting that ZDHHC11 must be a major target mRNA of *hnRNP G‐T*. Likewise, p‐ATM and p‐ATR were decreased by the downregulation of ZDHHC11 as well as hnRNP G‐T, suggesting that ZDHHC11 is key regulator of ATM/ATR signaling that mediates hnRNP G‐T. Therefore, hnRNP G‐T mediating the *ZDHHC11* mRNA pathway is a key pathway for the progression of CRC. However, the association between the remaining mRNAs and the tumor‐promoting effect of hnRNP G‐T also needs to be examined.

In RT‐PCR of clinical biopsy specimens, as shown in Figure 1C, the expression *hnRNP G‐T* showed individual differences in human CRC tissue, suggesting that the therapeutic effect may differ in each CRC patient when hnRNP G‐T is used as a therapeutic target. To apply hnRNP G‐T targeting therapeutics in CRC treatment, the development of biomarkers that are correlated with the effects of hnRNP G‐T targeting therapy may be needed. Likewise, we confirmed the tumor suppressive functions in three cancer cell lines: HCT116, SW480, and SUIT‐2. A previous investigation demonstrated that these cell lines have mutations in cancer driver genes, including KRAS.^36^, ^37^, ^38^, ^39^, ^40^, ^41^ The influence of genetic mutation of cancer driver genes by the downregulation of hnRNP G‐T in cancer cell treatment should be investigated.

In conclusion, we revealed that hnRNP G‐T was abnormally expressed in CRC and that it promoted tumor growth in CRC through ZDHHC11‐mediated inhibition of apoptosis. Importantly, a growth inhibition effect induced by the reduction of hnRNP G‐T was detected in cancer cells, but not non‐cancerous cells. Thus, hnRNP G‐T is an attractive therapeutic target for CRC.

## CONFLICT OF INTEREST

The authors declare no competing interest.

## AUTHOR CONTRIBUTIONS

Yuki Murakami, Hiroaki Konishi, and Mikihiro Fujiya contributed equally to this work. Yuki Murakami, Hiroaki Konishi, and Mikihiro Fujiya provided major input regarding the conceptual development of the studies, wrote the manuscript, and supervised all of the investigations. Yuki Murakami and Hiroaki Konishi performed the biochemical experiments. Yuki Murakami, Keitaro Takahashi, Katsuyoshi Ando, Nobuhiro Ueno, and Shin Kashima collected clinical biopsies. Kentaro Moriichi, Hiroki Tanabe, and Toshikatsu Okumura helped to design the studies, interpret the data, and prepare/review the manuscript. All of the authors read and approved the final manuscript.

## ETHICAL APPROVAL STATEMENT

This study was approved by the Medical Ethics Committee of Asahikawa Medical University (Approval number: 1668). The protocols of the animal experiments were approved by the Asahikawa Medical University Institutional Animal Care and Use Committee.

## Supporting information


Figure S1

Figure S2

Figure S3

Figure S4

Data S1

Table S1

Table S2

Table S3

Table S4

Table S5

Table S6

## Data Availability

The data sets used and analyzed during the current study are available from the corresponding author upon reasonable request. The Human Protein Atlas data in Figure S3 and S4 are available in structured XML format and can be downloaded from www.proteinatlas.org/about/download.
