# Supplementary figures and images for: Testis‐specific hnRNP is expressed in colorectal cancer cells and accelerates cell growth mediating ZDHHC11 mRNA stabilization
Source: Cancer Med. 2022 Apr 5;11(19):3643–56. doi: 10.1002/cam4.4738 (PMC9554453; doi:10.1002/cam4.4738)

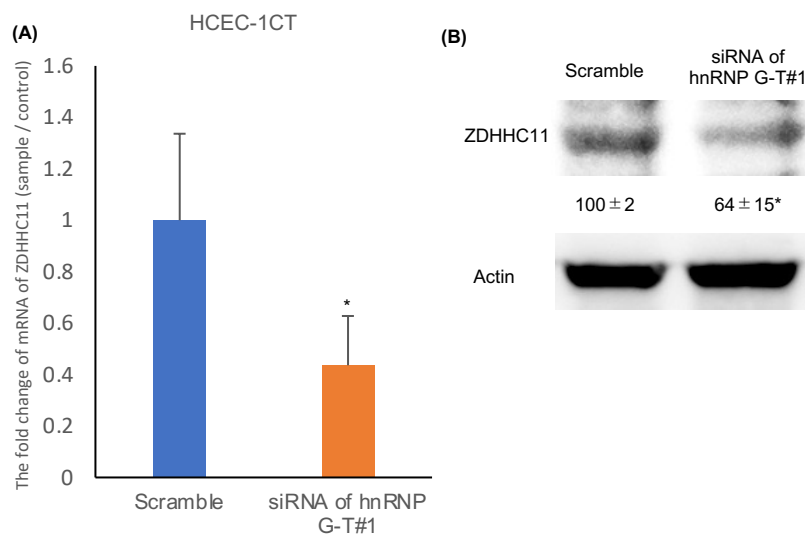

Figure.S1

Supplement: Supplementary file 1 — Figure S1 [file CAM4-11-3643-s003.pdf]

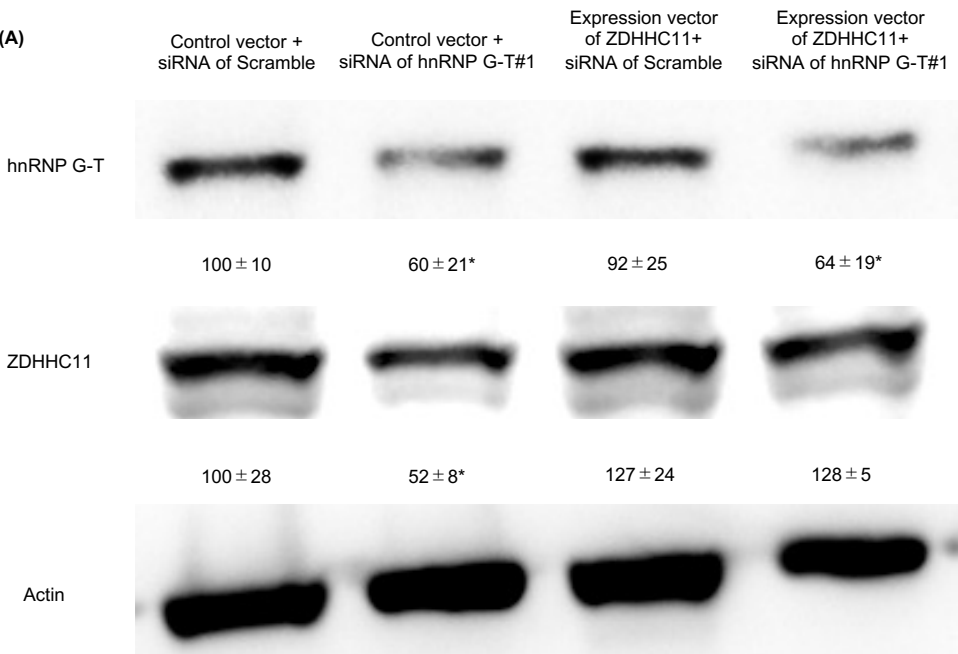

Figure.S2

Supplement: Supplementary file 2 — Figure S2 [file CAM4-11-3643-s008.pdf]

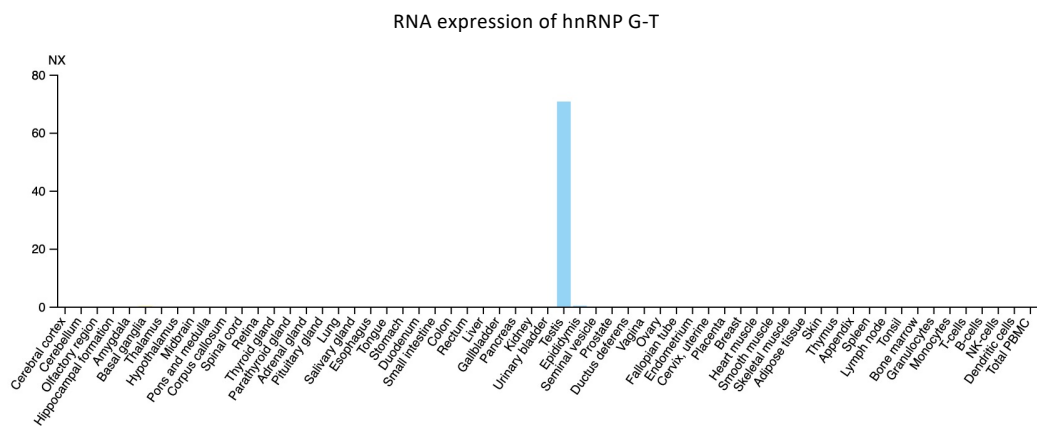

Figure.S3

Supplement: Supplementary file 3 — Figure S3 [file CAM4-11-3643-s006.pdf]

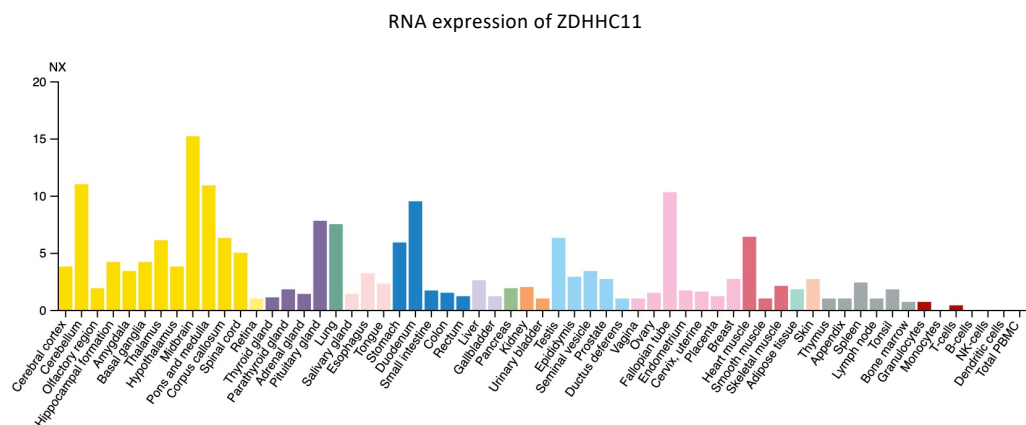

Figure.S4

Supplement: Supplementary file 4 — Figure S4 [file CAM4-11-3643-s010.pdf]
