# Supplementary material for: Testis‐specific hnRNP is expressed in colorectal cancer cells and accelerates cell growth mediating ZDHHC11 mRNA stabilization
Source: Cancer Med. 2022 Apr 5;11(19):3643–56. doi: 10.1002/cam4.4738 (PMC9554453; doi:10.1002/cam4.4738)
Supplement: Supplementary file 5 — Data S1 [file CAM4-11-3643-s001.docx]

**Supporting information figure legends**

**Figure S1. The expression of hnRNP G-T is correlated with the expression of ZDHHC11T in HCEC-1CT cells**

**A, B** RT-PCR (**A**) and Western blots (**B**) showed a decrease in the expression of *ZDHHC11* mRNA and protein in hnRNP G-T-downregulated HCEC-1CT cells (n=3).

**Figure S2. The expression of ZDHHC11 in hnRNP G-T-downregulated HCT116 cells recovered with the induction of the *ZDHHC11* expression vector.**

**A** Western blotting showed a decrease in the expression of ZDHHC11 protein in hnRNP G-T-downregulated HCT116 cells. The expression of ZDHHC11 recovered with the induction of the *ZDHHC11* expression vector (n=3).

**Figure S3. The RNA expression of *hnRNP G-T***

*hnRNP G-T* is specifically expressed in the testis in non-cancerous tissues. The expression data were downloaded from the Human Protein Atlas (www.proteinatlas.org/ENSG00000170748-RBMXL2/tissue).

**Figure S4. The RNA expression of *ZDHHC11***

*ZDHHC11* is expressed in many kinds of organs. The expression data were downloaded from the Human Protein Atlas (www.proteinatlas.org/ENSG00000188818-ZDHHC11/tissue).
